# Energy-efficient distributed heterogeneous re-entrant hybrid flow shop scheduling problem with sequence dependent setup times considering factory eligibility constraints

**DOI:** 10.1038/s41598-022-23144-6

**Published:** 2022-11-05

**Authors:** Kaifeng Geng, Li Liu, Zhanyong Wu

**Affiliations:** 1 Fan Li Business School, Nanyang Institute of Technology, Nanyang, 473004 Henan China; 2 School of Digital Media and Art Design, Nanyang Institute of Technology, Nanyang, 473004 Henan China

**Keywords:** Mechanical engineering, Computer science

## Abstract

In the face of energy crisis, manufacturers pay more and more attention to energy-saving scheduling. In the paper, we consider the distributed heterogeneous re-entrant hybrid flow shop scheduling problem (DHRHFSP) with sequence dependent setup times (DHRHFSP-SDST) considering factory eligibility constraints under time of use (TOU) price, which means that each job can only be assigned to its available set of factories and all factories have different number of machines and processing capacity, and so on. To deal with DHRHFSP-SDST, a multi-objective Artificial Bee Colony Algorithm (MOABC) is proposed to optimize both the makespan and total energy consumption. For the MOABC, firstly, a hybrid initialization method is presented to initialize the population; then, due to the electricity price shows significant differences vary from periods under TOU price, the energy saving operator based on right-shift strategy is proposed to avoid processing jobs with the high electricity price without affecting the productivity; thirdly, based on the full consideration of distributed heterogeneous and factory eligibility, crossover and mutation operators, three neighborhood search operators and new food sources generation strategy are designed; lastly, extensive experiments demonstrate the effectiveness of the proposed algorithm on solving the DHRHFSP-SDST.

## Introduction

Cooperative production among enterprises is becoming more common as globalization progresses. Therefore, enterprise managers decentralize production centers and transform them into distributed factories to reduce production costs, manage risks and improve enterprise competitiveness^1–3^. Hybrid flow shop scheduling problem (HFSP) widely exists in production practice, such as textile, semiconductor and paper industries^4^. It needs to solve two subproblems: machine selection and operation sequencing. Contrast to HFSP, the distributed hybrid flow shop problem (DHFSP) considers multiple factories, and it is an HFSP problem in each factory. As a result, DHFSP must address three subproblems: factory selection, machine selection and operation sequencing. DHFSP is currently being studied by researchers, and some preliminary findings have been produced. To solve the DHFSP, Shao et al.^5^ designed a hybrid algorithm based on DNEH and a multi-neighborhood iterated greedy algorithm to minimize makespan. Jiang et al.^6^ addressed the DHFSP using a novel evolutionary algorithm to minimize makespan and total energy consumption (TEC). Li et al.^7,8^ suggested two improved discrete artificial bee colony (DABC) algorithms for the DHFSP and DHFSP with sequence dependent setup times (SDST) to minimize makespan, separately. Meng et al.^9^ tackled the DHFSP with SDST using three mixed-integer linear programming (MILP) model. Cai et al.^10^ designed a VNS algorithm to minimize makespan and the total delay time for the DHFSP with SDST. Zheng et al.^11^ proposed an EDA algorithm based on iterated greedy search to solve the multi-objective fuzzy DHFSP.

However, most of the previous studies assume that the factory is identical in the distributed scheduling problem, and lack of consideration for the constraint of heterogeneous factory. In addition, most of the optimization objectives are time related indicators, while there are few environmental related indicators such as energy consumption. Lu et al.^12^ addressed the distributed heterogeneous flow shop scheduling problem (DHFSP) using a hybrid algorithm. Focus on the DHFSP, an evolutionary algorithm is designed to minimize the makespan and TEC^13^. Liu et al.^14^ proposed a DABC algorithm for the distributed heterogeneous scheduling problem (DHSP). Focus on the no-wait DHFSP, a DABC algorithm is designed to minimize makespan^15^. Zhao et al.^16^ designed a self-learning discrete yaya algorithm to solve the no-idle DHFSP to minimize the total tardiness (TD), TEC, and factory load balancing. In recent years, environmental issues have gotten a lot of attention. Reducing carbon emissions and developing green economy have become the consensus of all countries in the world. Green scheduling, as we all know, is a strategy for conserving energy and lowering emissions. For the energy-efficient scheduling problem, He et al.^17^ studied the energy-efficient job shop scheduling problem with SDST to minimize makespan, TD and TEC. Pan et al.^18^ provided a bi-population evolutionary algorithm to solve the energy-efficient fuzzy flexible job shop scheduling problem (FJSP). Wang et al.^19^ designed a whale swarm algorithm for the distributed welding flow shop scheduling problem aiming at minimizing the TEC and makespan. Focus on the energy-efficient distributed permutation flow-shop inverse scheduling problem, a hybrid collaborative algorithm is designed^20^. Lian et al.^21^ studied the energy-efficient HFSP in steelmaking plants. For convenience, these above publications are classified by shop floor category, objectives, solving approach (algorithms), (see Table 1).

**Table 1:** Literatures classification.

Articles	Shop floor category	Objectives	Approach (algorithm)
Lu et al.^12^	Distributed heterogeneous scheduling problem	Makespan and TEC	Iterated greedy algorithm
Wang et al.^13^	Distributed heterogeneous welding flow shop	Makespan and TEC	MOEA/D algorithm
Liu et al.^14^	Distributed heterogeneous integrated process planning and scheduling problem	Makespan	DABC algorithm
Haoran et al.^15^	Distributed heterogeneous no-wait flowshop scheduling problem	Makespan	DABC algorithm
Zhao et al.^16^	Distributed Heterogeneous No-Idle Flow-Shop Scheduling Problem	TD, TEC, and factory load balancing	Discrete yaya algorithm
He et al.^17^	Job shop scheduling problem with SDST	Makespan, TD and TEC	Hybrid multiobjective genetic algorithm
Pan et al.^18^	Fuzzy flexible job shop scheduling problem	Fuzzy makespan and fuzzy TEC and maximize minimum agreement index	Bi-population evolutionary algorithm
Wang et al.^19^	Distributed welding flow shop scheduling problem	Makespan and TEC	Whale swarm algorithm
Mou et al.^20^	Distributed permutation flow-shop inverse scheduling problem	Minimize adjustment and TEC	Hybrid collaborative algorithm
Lian et al.^21^	Hybrid flow shop scheduling problem	Makespan and TEC	Improved multi-objective evolutionary algorithm

In practice, some machines are new and some ones are old in the same factory, the processing capacity varies from machines, and some jobs cannot be processed on all factories. If there is a machine in a factory that is not suitable for one job, the job cannot be arranged to the factory, namely factory eligibility. In addition, statistics^22^ show that: in actual production, non-processing time accounts for more than 90% of the whole production time. Therefore, auxiliary time such as setup times can not be ignored. However, in classical job scheduling optimization problems, setup time is usually considered in processing time or ignored. At present, no published relevant research results have been found about the distributed heterogeneous re-entrant hybrid flow shop scheduling problem with sequence dependent setup times considering factory eligibility constraints (DHRHFSP-SDST) under TOU price. As a result, this article focused on the DHRHFSP-SDST, which introduces new economic and environmental dimensions to this problem and realizes green production. The following are the primary contributions of this paper:

1. Aiming at the DHRHFSP-SDST, the MOABC algorithm is proposed to solve it. Meanwhile, crossover and mutation operators, three neighborhood search operators and new food sources generation strategy are designed to improve the performance of the algorithm.
2. According to the characteristic that the electricity price varies from hour to hour in one day, the implementation of green scheduling under TOU price is studied.
3. Considering the factory eligibility and SDST constraints, the effective factory allocation method, encoding and decoding strategy are designed.

The rest of the article is structured as follows: In “Problem description” section, the DHRHFSP-SDST is described; In “Methods” section, a MOABC algorithm is presented. In “Simulation experiment” section, some simulation experiments and the discussions are carried out. Finally, some conclusion and future works are discussed.

## Problem description

**Figure 1:**
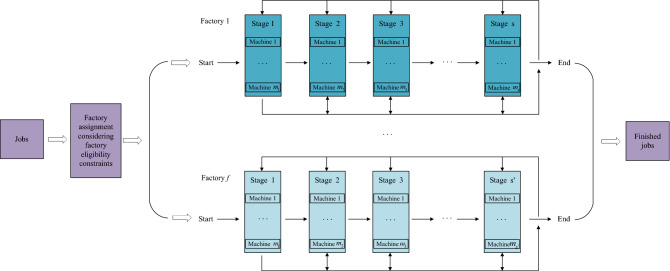
The production model of DHRHFSP-SDST.

DHRHFSP-SDST can be described as follows. *n* jobs need to be processed in *F* heterogeneous factories and each factory has an only one HFSP that has the same amount of stages. At each stage, there is one or more unrelated parallel machines (UPM). All jobs have the similar process routes, but some jobs may visit the same stage many times and can skip certain stages until all operations are completed. Each job has at least one factory that can process it. Due to the different quality, quantity and condition of machines in factories, some jobs cannot be processed on all machines. If there is a machine in a factory that is not suitable for one job, the job cannot be arranged to the factory, namely factory eligibility^23^. The setup time of an operation cannot be ignored, and it is related to machine and the adjacent operations, namely SDST. The aim of this problem is to allocate all jobs to the available factories reasonably and determine the processing sequence in each factory, so as to minimize the makespan and total energy cost considering factory eligibility and sequence dependent setup times. Figure 1 is the production model of the DHRHFSP-SDST.

The main assumptions for DHRHFSP-SDST are as follows:

1. Each job can be allocated to just one of the factories that are available and the processing time of the same operation varies from machines and factories.
2. The number of UPMs in each factory varies, but the number of stages remains the same.
3. The first operation's setup time on each machine is not considered.
4. Once a job has been assigned to a factory, all of its operations should be handled at that factory.
5. The processing time and setup time of all operations are known, the unit energy consumption in the processing state, idle state and setup state are known, and the TOU price scheme is known.
6. Regardless of the machine failure, the energy consumption of power on/off, the buffer between adjacent machines is infinite.

## Notations

**Table:** 

Notation	Description
*F*	Number of factories
*f*	Index for factories, *f* = 1, 2, …, *F*
*j,h,v*	Index for job, *j,h,v* = 1, 2, …, *n*
*L*	A large positive number
$s$	Number of stages in each factory
$i$	Index for stage,$i$ = 1, 2, …, *s*
*r*	Number of re-entrances
$k$	Index of operations for job *j*, $k \le r*s$
*O*_*jk*_	The *k-th* operation of job *j*
$m_{if}$	Number of UPMs at stage *i* in factory *f*
*u*	Index for UPM at stage *i* in factory *f*,* u* = 1, 2, …,$m_{if}$
$M$	The total number of machines in all factories, $M = \sum\limits_{f = 1}^{F} {\sum\limits_{i = 1}^{s} {m_{if} } }$
$q$	Index of machines,$q$ = 1, 2, …, *M*
$U_{if}$	Operations set that are processed at stage *i* in factory *f*
$S_{jkf}$	Starting time of $O_{jk}$ in factory *f*
$E_{jkf}$	Ending time of $O_{jk}$ in factory *f*
$p_{jkuif}$	Processing time of $O_{jk}$ on machine *u* at stage *i* in factory *f*
$u_{jhuif}$	Sequence dependent setup time of job *h* when job *j* and *h* are processed successively on machine *u* of station *i* in factory *f*
*C*_*j*_	Job *j*’s finished time
*S*_*q*_	Machine *q’*s startup time
*T*_*q*_	Machine *q’*s showdown time
*PI*_*q*_	Energy consumption per unit time of machine *q* in the idle state
*PW*_*q*_	Energy consumption per unit time of machine *q* in the processing state
*PS*_*q*_	Energy consumption per unit time of machine *q* in the setup state
$f(t)$	TOU price function
*Y*_*jf*_	1 if job *j is* assigned to factory *f*, and 0 otherwise
*Q*_*if*_	1 if job *j* can be processed in factory *f*; 0 otherwise
*X*_*jkuig*_	1 if *O*_*jk*_ is assigned to machine *u* at stage* i* in the factory *g,* and 0 otherwise
$Z_{{jkj^{^{\prime}} k^{^{\prime}} uif}}$	1 if *O*_*jk*_ precedes $O_{{j^{\prime } k^{\prime } }}$ are processed successively on machine *u* of station *i* in factory *f*, and 0 otherwise
$y_{q}^{t}$	1 if the machine *q* is in the processing state at time *t*, and 0 otherwise
$x_{q}^{t}$	1 if the machine *q* is in the idle state at time *t*, and 0 otherwise
$\lambda_{q}^{t}$	1 if the machine *q* is in the setup state at time *t*, and 0 otherwise

### Optimized objectives

Based on the existing literatures^1,2^, a mathematical model for bi-objective DHRHFSP-SDST is presented to minimize the makespan (*C*_*max*_) and total energy cost (TC). In real manufacturing, if the machines utilized for each operation varies, the processing time and energy usage differ. Similarly, different time periods are selected for processing, and the corresponding electricity price is different, so the processing energy consumption cost is also different. If the electricity price peak is avoided and the processing is selected in the trough period, the processing energy consumption cost will be reduced, but the standby energy consumption cost may increase and the maximum completion time may be extended due to the delay.

$$f = minimize(C_{max} ,TC)$$

1 $$C_{max} = max(C_{j} )$$

2 $$TC = PC + IC + SC$$

In which, *PC*, *IC*, and *SC* represent the energy cost of machines in processing, idle, and setup states, respectively.

3 $$PC = \sum\limits_{q = 1}^{M} {\sum\limits_{{t = S_{q} }}^{{T_{q} }} {PW_{q} y_{q}^{t} } f(t)}$$

4 $$IC = \sum\limits_{q = 1}^{M} {\sum\limits_{{t = S_{q} }}^{{T_{q} }} {PI_{q} x_{q}^{t} } } f(t)$$

5 $$SC = \sum\limits_{q = 1}^{M} {\sum\limits_{{t = S_{q} }}^{{T_{q} }} {PS_{q} \lambda_{q}^{t} } } f(t)$$

s.t.

6 $$\sum\limits_{f = 1}^{F} {Y_{jf} = 1} ,\quad \forall \, j$$

7 $$Y_{if} \le Q_{if} ,\quad \forall j,f$$

8 $$y_{q}^{t} + x_{q}^{t} + \lambda_{q}^{t} = 1,\quad \forall q,t = [S_{q} ,T_{q} ]$$

9 $$\sum\limits_{u = 1}^{{m_{if} }} {X_{jkuif} } = 1,\quad \forall \, i,j,k,f,\,O_{jk} \in U_{if}$$

10 $$\sum\limits_{u = 1}^{{m_{{i^{^{\prime}f} }} }} {r_{{j(k + 1)ui^{^{\prime}} f}} S_{j(k + 1)f} } \ge \sum\limits_{{u^{^{\prime}} = 1}}^{{m_{if} }} {r_{{jku^{^{\prime}} if}} (S_{jkf} + p_{{jku^{^{\prime}} if}} )} ,\quad \forall i,i^{^{\prime}} ,j,k,f$$

11 $$L(1 - X_{jkuif} ) + L(1 - X_{{j^{^{\prime}} k^{^{\prime}} uif}} ) + L(1 - Z_{{jkj^{^{\prime}} k^{^{\prime}} uif}} ) + (S_{{j^{^{\prime}} k^{^{\prime}} f}} - S_{jkf} ) \ge p_{jkuif}\, + u_{{jj^{^{\prime}} uif}} ,\quad \forall i,j,j^{^{\prime}} ,u,O_{jk} \in U_{if} ,O_{{j^{^{\prime}} k^{^{\prime}} }} \in U_{if}$$

12 $$L(1 - X_{jkuif} ) + L(1 - X_{{j^{^{\prime}} k^{^{\prime}} uif}} ) + LZ_{{jkj^{^{\prime}} k^{^{\prime}} uif}} + (S_{jkf} - S_{{j^{^{\prime}} k^{^{\prime}} f}} ) \ge p_{{j^{^{\prime}} k^{^{\prime}} uif}} + u_{{j^{^{\prime}} juif}} ,\quad \forall i,j,j^{^{\prime}} ,u,O_{jk} \in U_{if} ,O_{{j^{^{\prime}} k^{^{\prime}} }} \in U_{if}$$

13 $$L(1 - X_{jkuif} ) + L(1 - X_{{jk^{^{\prime}} uif}} ) + (S_{{jk^{^{\prime}} f}} - S_{jkf} ) \ge p_{jkuif} ,\quad \forall i,u,j,f,k < k^{^{\prime}} ,O_{jk} \in U_{if} ,O_{{jk^{^{\prime}} }} \in U_{if}$$

14 $$L(4 - X_{{j(k - 1)u^{^{\prime}} i^{^{\prime}} f}} - X_{jkuif} - X_{{hk^{^{\prime}} uif}} - Z_{{hk^{^{\prime}} jkuif}} ) + S_{jkf} \ge max(E_{{hk^{^{\prime}} f}} + u_{{hk^{^{\prime}} jkuif}} ,E_{j(k - 1)f} ),\quad \forall j,h,k^{^{\prime}} ,u,u^{i} ,i,i^{^{\prime}} ,f,k > 1$$

15 $$E_{jkf} \le S_{jkf} { + }p_{jkuif} + L(1 - X_{jkuif} ),\quad \, \forall \, j,k,u,i,f$$

16 $$C_{j} \le C_{max} ,\quad \forall \, j$$

17 $$C_{j} \ge 0,\quad \forall \, j$$

Formulas (1–2) are the objective functions. Formulas (3–5) are the three components of *TC.* Constraint (6) requires that each job be allocated to just one factory. Constraint (7) indicates that each job must be arranged to a factory that can process it. Constraint (8) indicates that in the processing cycle, each machine only has three states: processing, idle and setup. Constraint (9) assures that each operation will only be processed on one machine at one stage. Constraint (10) assures that the starting time is not earlier than the prior operation's finishing time. Constraints (11–13) ensure that each machine can only process one operation at a time. Constraints (14–15) indicate the $S_{jkg}$ and $E_{jkg}$. Constraints (16–17) indicate the $C_{j}$.

## Methods

The DHRHFSP-SDST is an NP hard problem that is difficult to solve using exact approaches. Metaheuristic algorithms have a significant benefit in terms of balancing computation time and solution quality. The artificial bee colony algorithm (ABC) is a novel intelligent optimization algorithm based on the honeybee's honey harvesting process, which has three major components: food source, employed bees and unemployed bees^24^. The food sources are the feasible solution of the optimization problem to be solved and the quality of the food sources or the feasible solutions is evaluated by the amount of nectar of nectar sources, namely the fitness. The number of employed bees or onlooker bees is equal to the number of food sources. The task of the employed bees is mainly to find information about food sources and share it with the onlooker bees with a certain probability. Unemployed bees include onlooker bees and scout bees. After receiving the information transmitted by the employed bees, the onlooker bees chose the satisfactory nectar source greedily for tracking. If a food source has not been further updated after *limit* times cycle, the onlooker bee will become a scout bee, and the scout bee will find a new food source instead of the original one.

**Figure 2:**
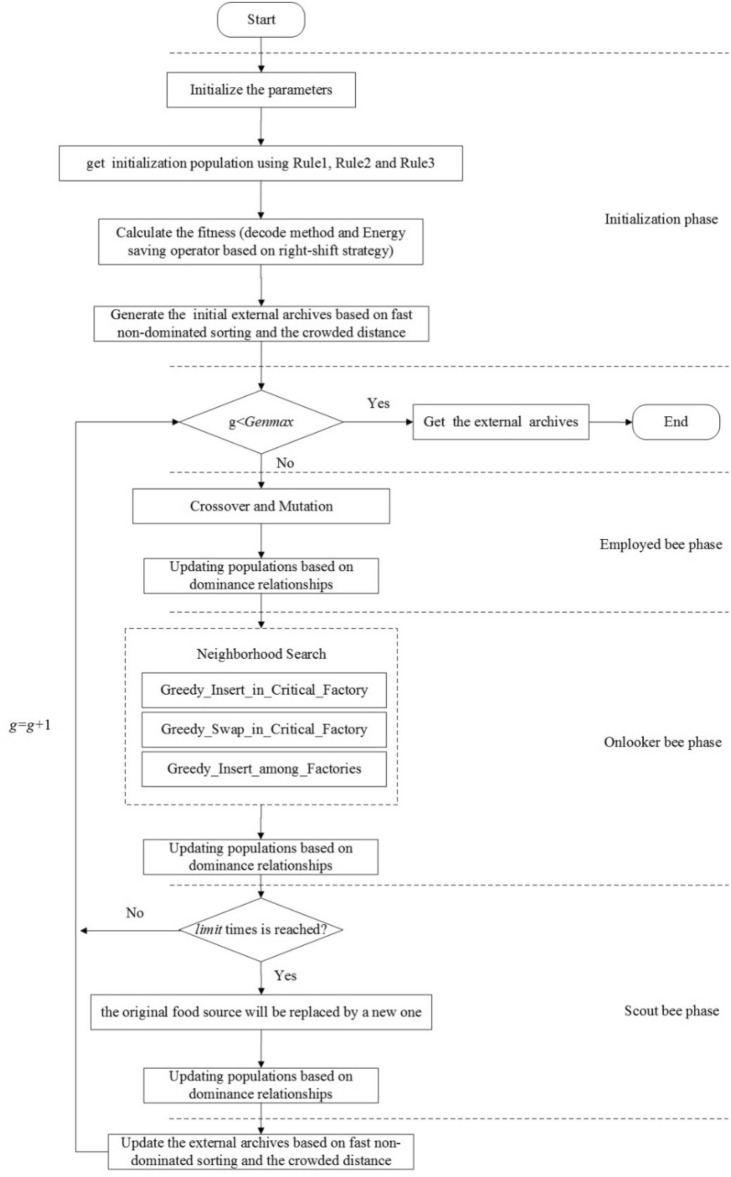
Flow chart of MOABC algorithm.

As shown in Fig. 2, the improved MOABC algorithm proposed. A hybrid initialization method is presented to initialize the population; then, due to the electricity price shows significant differences vary from periods under TOU price, the energy saving operator based on right-shift strategy is proposed to avoid processing jobs with the high electricity price without affecting the productivity; lastly, based on the full consideration of distributed heterogeneous, factory eligibility and SDST features, crossover and mutation operators, three neighborhood search operators and new food sources generation strategy are designed.

### Individual expression

**Figure 3:**
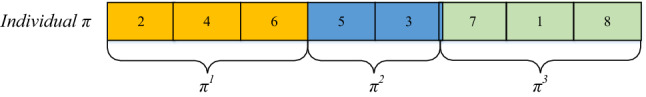
Individual expression.

In the MOABC algorithm, each feasible solution represents a food source. According to the characteristics of DHRHFSP-SDST, we employ the representation as in Naderi and Ruiz^25^ for the classic distributed permutation flow shop scheduling problem. To be more descriptive, the individual is represented by *f* vectors, one for each factory. Each vector is a job list that represents the jobs sequence processed in the factory. An individual may be written as *π* = {*π*^*1*^, *π*^*2*^, … , *π*^*F*^} using the aforementioned encoding approach, in which *π*^*f*^ denotes the permutation that is the order of jobs processed in factory *f,* as shown in Fig. 3.

### Decoding method

**Figure 4:**
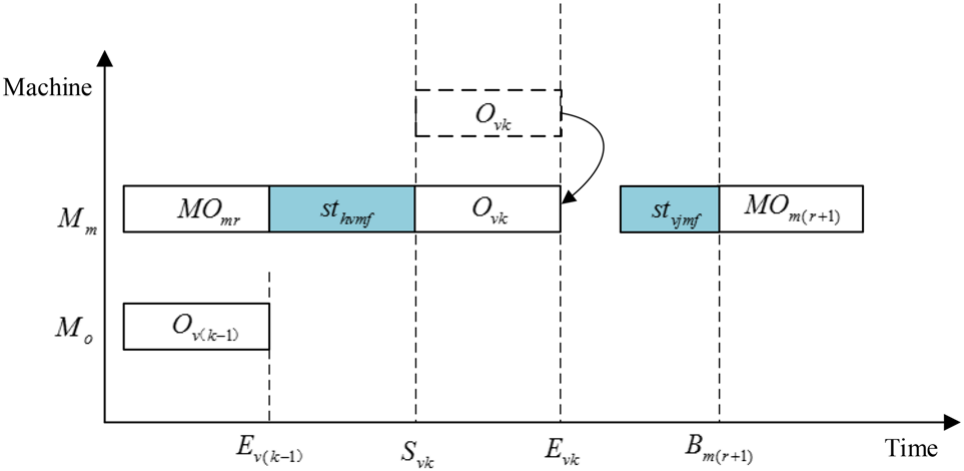
The decoding diagram of operation $O_{vk}$ when $E_{vk} \le B_{{m ( r{ + }1 ) }} - st_{vjmf}$.

**Figure 5:**
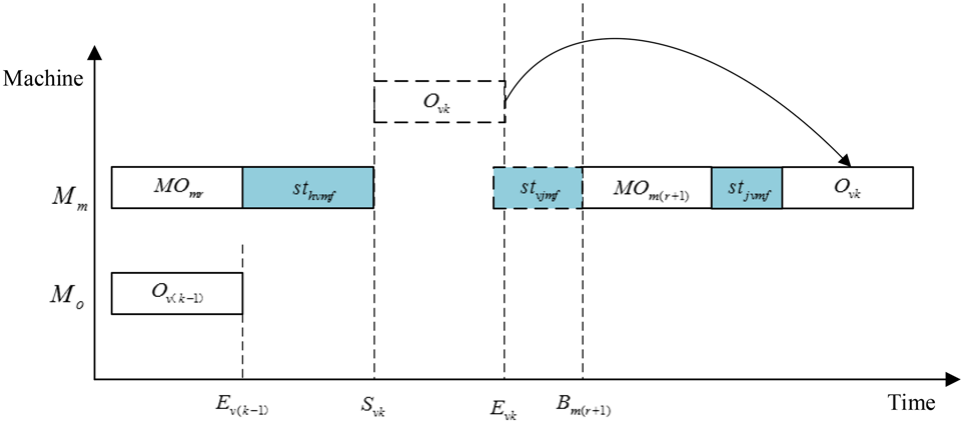
The decoding diagram of operation $O_{vk}$ when $E_{vk} > B_{{m ( r{ + }1 ) }} - st_{vjmf}$.

Assign the jobs to the appropriate factory according to the encode schema and, if possible, place it on a machine that can process it as soon as possible. The decoding process is shown in Figs. 4 and 5, and the detailed decoding steps are as follows.

*Step 1* Assign all jobs to corresponding factories according to individual π and initialize *f* = 1, *index* = 1.

*Step 2* Traverse all of the jobs in factory *f* and set *joblist* = $\pi^{f}$.

*Step 3* Assuming $v = joblist(index)$, if job *v* is the first job processed in the factory *f*, all operations of job *v* will be arranged on the earliest machine that can complete the operation, and the starting and ending times of each operation will be recorded, and then turn to step 8, otherwise turn to step 4.

*Step 4* From the available machines set *machineset*, choose the appropriate machine for operation $O_{vk}$.

*Step 5* Assume that machine *u* is selected from *machineset* to process operation $O_{vk}$. Then the starting time $S_{vkf}$ and ending time $E_{vkf}$ of operation $O_{vk}$ can be obtained by the following formulas.

18 $$S_{vkf} = max\{ E_{v(k - 1)f} ,p_{u} + u_{hvuif} \}$$

19 $$E_{vkf} = S_{vkf} { + }p_{vkuif}$$

where $E_{v(k - 1)f}$ represents the ending time of the previous operation of job *v*. If operation $O_{vk}$ is the first operation of job *v*, then $E_{v(k - 1)f} { = }0$. $p_{u}$ refers to the ending time of an operation on machine $u$. $u_{hvuif}$ is the SDST of operation $O_{vk}$. If $O_{vk}$ is the first operation of machine *u*, then $u_{hvuif} { = }0$.

*Step 6 S*elect an appropriate insertion point for operation $O_{vk}$ on machine $u$ of factory *f* to minimize $S_{vkf}$. The detailed steps are as follows:

1. Obtain all the operation sets *ops* in the current state of machine *u*, and arrange them in ascending order according to the ending time.
2. Get the ending time $C_{mr}$ of the *r*-*th* operation in *ops*. $p_{u} = C_{mr}$, where *r* = 0 means that operation $O_{vk}$ is regarded as the first operation of the equipment, and $p_{u} = C_{mr} = 0$.
3. According to the formulas (18) and (19), the starting time and ending time of operation $O_{vk}$ are calculated. If the *r*-*th* operation is the last operation on machine *u*, return $S_{vkf}$ and $E_{vkf}$, and end the judgment. Otherwise, if $E_{vkf} \le B_{{u(r{ + }1)f}} - u_{vjuif}$, where $B_{{u(r{ + }1)f}}$ is the starting time of the (*r* + *1*)*-th* operation on the machine *u*, return $S_{vkf}$ and $E_{vkf}$, and end the judgment. Otherwise,* r* = *r* + 1, go to (2).

*Step 7* Repeat Step 6 until all the machines in *machineset* are traversed to get the smallest $S_{vkf}$. If multiple insertion points have the same $S_{vkf}$, select one with smaller idle time between the insertion point and the previous operation.

*Step 8 index* = *index* + 1, repeat Step 3—Step 7 until all jobs in $joblist$ are processed.

*Step 9 f.* = *f* + 1, go to step 2 until all factories are traversed.

*Step 10* Calculate the objective function values *C*_*max*_ and *TC.*

Consider the following scenario: 2 factories, 8 jobs and 2 stages. The number of UPMs at each stage in factory1 is 2 and 2. The amount of UPMs at each stage in factory2 is 1 and 2. The factory eligibility constraints is F_1_ = {1,2}, F_2_ = {1}, F_3_ = {1,2}, F_4_ = {2}, F_5_ = {1,2}, F_6_ = {1}, F_7_ = {1,2}, F_8_ = {1,2}, which indicates that job 1, job3, job5, job7 and job 8 can be processed in all factories, job 2 and job6 can be processed in factory 1 only and job4 can be processed in factory2 only. To make things more understandable, assume that *π* = {[1–8]} with *π*^*1*^ = [1, 2, 6–8] and *π*^*2*^ = [3–5], which means that job1, job 2, job6, job7 and job8 are processed in factory1 in the order job1 → job 2 → job6 → job7 → job8, while job3, job4 and job5 are processed in factory2 in the order job3 → job4 → job5. For instance, in factory1, all of job 1's operations are scheduled on the machine that can complete them the earliest. Then, all operations of the rest jobs will be placed on the machines that can complete them the earliest by selecting the proper insertion points. The processing times of 8 jobs in each available factory is shown in Table 2. $p_{vk}$ = 0 means that the job is not machined at this stage in this round. Meanwhile, Tables 3 and 4 show the sequence dependent setup times and the TOU price scheme, respectively.

**Table 2:** Processing times.

Factories	Jobs	First-round				Re-entrant 1			
		Stage1		Stage2		Stage1		Stage2	
		M1	M2	M3	M4	M1	M2	M3	M4
Factory 1	Job 1	3	5	5	5	4	3	1	1
	Job 2	1	3	2	2	5	5	5	3
	Job 6	2	1	3	4	5	4	3	5
	Job 7	2	2	3	1	5	3	3	3
	Job 8	5	5	0	0	5	2	3	4

		M1		M2	M3	M1		M2	M3
Factory 2	Job 3	2		3	3	1		0	0
	Job 4	4		2	5	1		5	4
	Job 5	2		2	5	4		3	1

**Table 3:** Sequence dependent setup times.

Factories	Stages	Machines	Jobs	Job 1	Job 2	Job 6	Job 7	Job 8
Factory 1	Stage 1	M1/M2	Job 1	0/0	3/2	3/3	2/3	1/1
			Job 2	3/3	0/0	3/2	2/2	3/1
			Job 6	2/1	1/1	0/0	1/3	3/3
			Job 7	3/3	1/1	3/2	0/0	1/1
			Job 8	3/3	3/2	3/3	1/1	0/0
	Stage 2	M3/M4	Job 1	0/0	2/2	1/3	1/1	2/1
			Job 2	3/1	0/0	1/3	3/3	3/1
			Job 6	1/1	1/3	0/0	2/2	2/2
			Job 7	2/3	3/1	3/3	0/0	3/1
			Job 8	2/3	1/1	2/1	3/3	0/0

				Job3	Job 4	Job 5		
Factory 2	Stage 1	M1	Job 3	0	3	3		
			Job 4	1	0	1		
			Job 5	2	1	0		
	Stage 2	M2/M3	Job 3	0/0	2/2	1/1		
			Job 4	2/2	0/0	2/2		
			Job 5	3/1	1/2	0/0

**Table 4:** TOU price scheme (unit: CNY/kWh).

	On-peak	Mid-peak	Off-peak
Time	8:00–11:00 13:00–1500	6:00–8:00 11:00–13:00 15:00–22:00	0:00–6:00 22:00–24:00
Price	1.202	0.749	0.285

**Figure 6:**
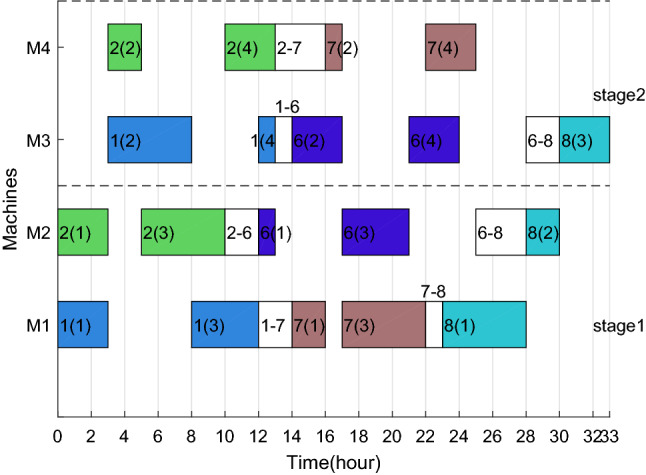
Gantt chart of factory1.

**Figure 7:**
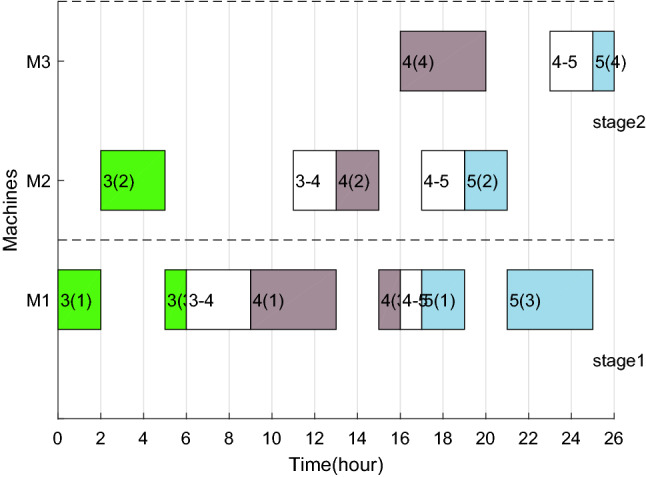
Gantt chart of factory2.

The Gantt charts obtained according to the above decoding method are shown in Figs. 6 and 7. Among them, 1(1) denotes the first operation of job 1, and in addition, 1–3 on machine 2 denote the sequence dependent setup times between job 1 and job 3 on machine 2. The $C_{max}$ and *TC* in each factory are 33 h, 524.896 CNY and 26 h, 261.656 CNY respectively. Therefore, the two objective values are $C_{max} { = }$ *max*(33,26) = 33 h and $TC{ = }$ 524.896 + 261.656 = 786.552 CNY.

### Energy saving operator based on right-shift strategy

Because waiting states for machines and jobs are unavoidable in SRHFSP-SDST, we can make full use of these waiting periods by adjusting the processing times of the operations to reduce the *TC*. Based on the factory assignment, job sequencing and machine assignment, the energy saving operator based on right-shift strategy (ESRS) is added to select the appropriate starting processing time for each operation and try to avoid processing jobs in the periods with high electricity prices to achieve a lower *TC* without deteriorating *C*_*max*_. According to various constraints, all operations can only be shifted to the right. Furthermore, the adjustment of the latter operation will affect the previous operation and only machines with idle periods have the potential to shift to right, so the right shift procedure must be carried from back to front. The specific steps of ESRS is as follows:

*Step 1* Input a solution. Generate a scheduling scheme according to the decoding method, and record the starting time and ending time of all operations, the starting time and ending time of all setup periods and initialize *f* = 1.

*Step 2* Put the starting time and ending time of all idle time periods of machines in plant *f* into the set $idle$, and record the information about the previous and the next operations of all the idle periods. If there is only one operation $O_{vk}$ on the machine and its completion time is less than *C*_*f*_ , then the time period [$E_{vk}$, $C_{f}$] is added to the *idle*.

*Step 3* Sort the idle periods in *idle* in descending order by the ending time of each idle period.

*Step 4* Traverse all idle periods in *idle*. Suppose that the idle time period is $\left[ {start,end} \right]$ and the previous operation is $O_{vk}$, then the ending time of $O_{vk}$ after the right-shift is $\overline{{E_{vk} }} = min(end,S_{vk + 1} )$. If $O_{jk}$ is the last operation of job *v*, then $\overline{{E_{vk} }} = C_{f}$. If $E_{jk} < \overline{{E_{jk} }}$, it means that operation $O_{vk}$ meets the condition of shift to right, and the step size is $\overline{{E_{vk} }} - E_{vk}$. Meanwhile, adjust the operation's processing and setup times and move to step 2. If $E_{jk} { = }\overline{{E_{jk} }}$, continue with the next idle period until the *idle* is traversed.

*Step 5 f.* = *f* + *1*, repeat Step2–Step5 until all factories are traversed.

*Step 6* Generate a new scheduling scheme.

**Figure 8:**
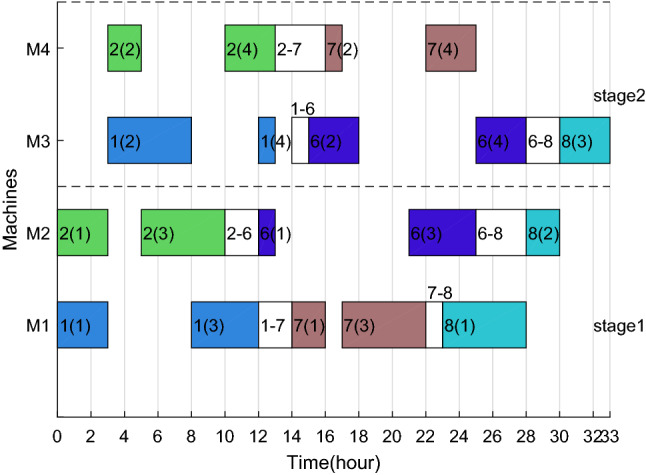
Factory1's Gantt chart with ESRS.

**Figure 9:**
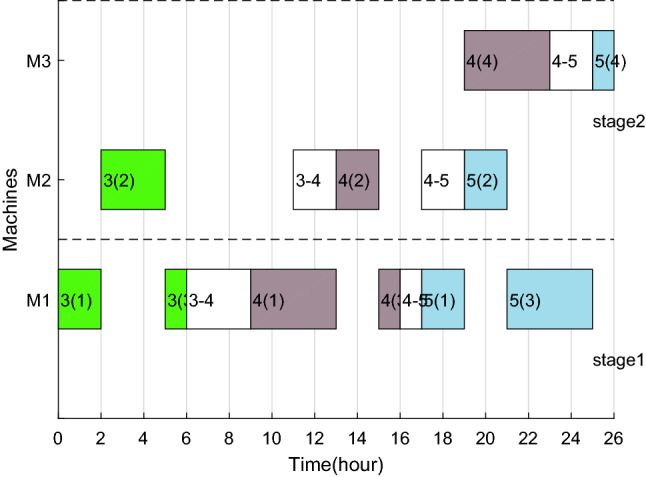
Factory2's Gantt chart with ESRS.

After adding ESRS to the scheduling scheme shown in Figs. 6, 7, 8 and 9 illustrate the revised Gantt charts with the objective values $C_{max} { = }$ 33 h and $TEC{ = }$ 751.366 CNY. By comparison, *TC* was reduced by 35.186 CNY, up to 4.47%, without affecting $C_{max}$. The reason is that the processing periods of operations 6(2), 6(4) and 4(4) are shifted from the period with high electricity price to low electricity price.

**Figure 10:**
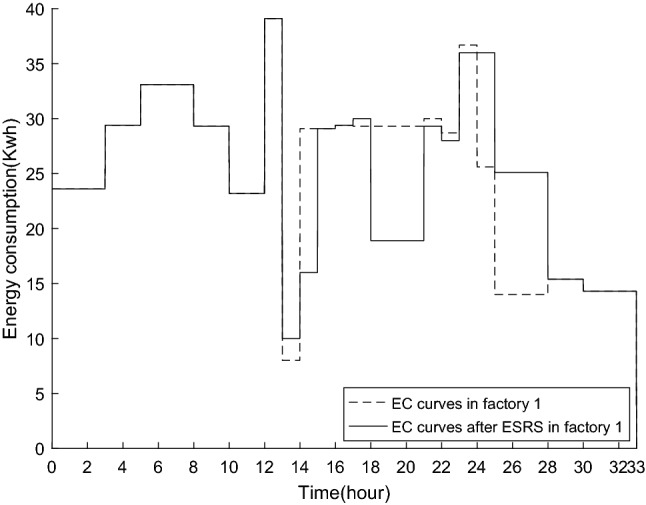
The EC curve in factory1.

**Figure 11:**
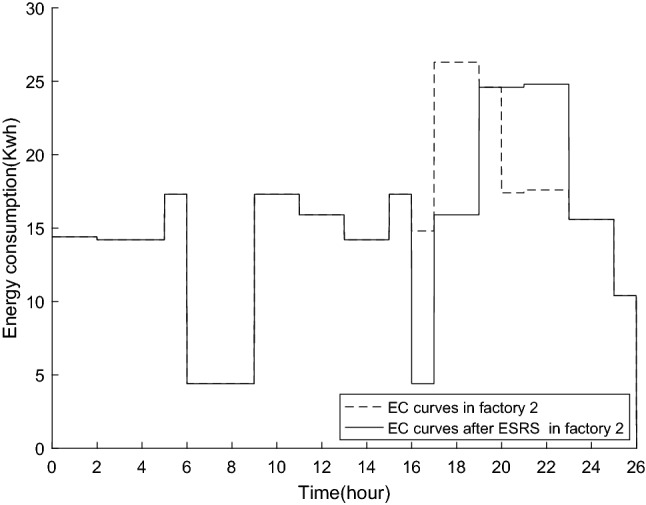
The EC curve in factory2.

Figures 10 and 11 show the energy consumption curve (EC curve) of all machines in each factory. As seen in the figures, if there is a discrepancy between the dotted and solid lines, the electrical load has transferred. In particular, electricity is charged at a high (low) price in the vicinity of 18–21 (25–28) hours in factory1. After ESRS, the TC in the first phase drops, whereas the TC in the second period rises. Factory2 is similar to that in factory1. Evidently, total energy costs can be reduced by avoiding higher price periods.

### Population initialization

The quality of the initial population has a direct impact on the performance of the MOABC algorithm. According to the characteristics of solving problem and various constraints, three kinds of population initialization methods are designed in this paper. To guarantee the population's variety, the Randomly_initialization method is used for 20% of the population. In addition, to ensure the quality of the solutions, 40% individuals are generated equitably based on two greedy methods: makespan_based_initialization and TC_based_initialization.
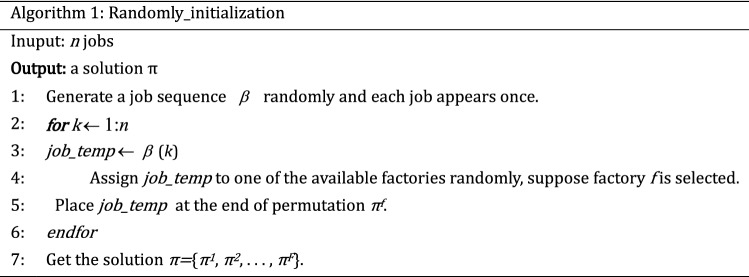



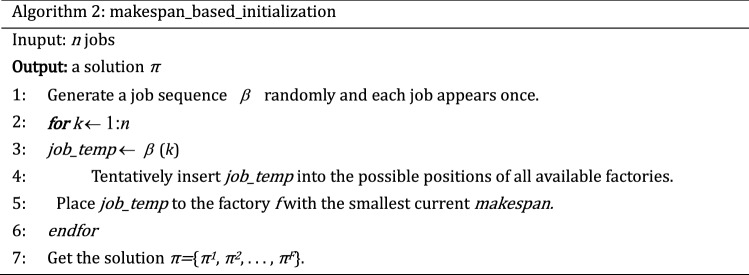



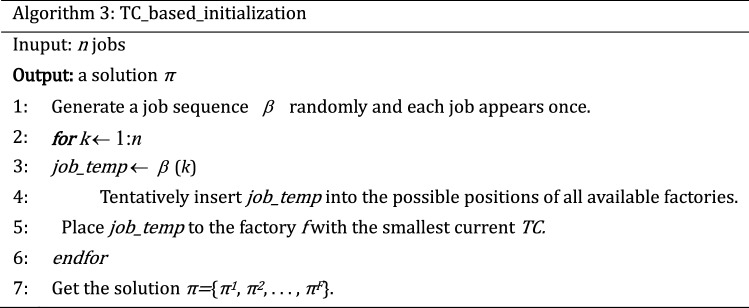


### Employed bee phase

**Figure 12:**
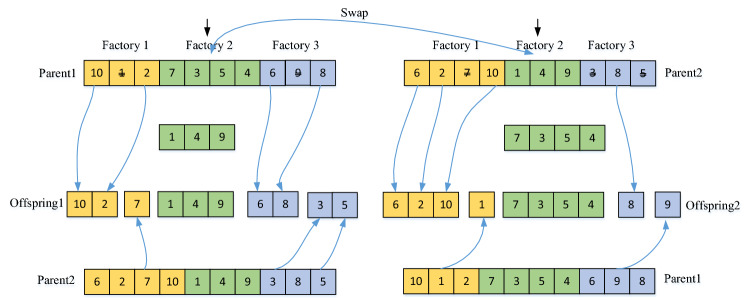
Crossover operator.

Crossover and mutation operators are used in the employed bee phase to enhance the population's diversity. Crossover is an indispensable part in the evolutionary process, which generates new individuals by exchanging genes between two different parental individuals. According to the characteristics of the encoding and the factory eligibility constraints, this paper adopts the crossover operator as shown in Fig. 12. First, select factory *f* randomly and swap the jobs in factory *f* between two parent individuals. Then, remove the jobs duplicated with factory *f* in Parent1 and Parent 2. Thirdly, copy the remaining jobs in Parent1 into offspring 1 and the remaining jobs in Parent 2 into offspring 2. Lastly, copy part of the jobs in Parent 2 into offspring 1 and part of the jobs in Parent 1 into offspring 2.

To avoid that the better food source obtained after the crossover operate is not destroyed, the following selection strategy is designed. If O1 and O2 are generated by the crossover operator performed on the parent food sources P1 and P2. Select a better one as *X*_*better*_*.* If *f*(*X*_*better*_)$\prec$ *f(P1*), then P1 $\leftarrow$ *X*_*better*_ and *limit* = 0 else if *f*(*X*_*better*_)$\prec$ *f(P2*), then P2 $\leftarrow$ *X*_*better*_ and *limit* = 0; else perform the following mutation operator. To avoid the infeasible solution caused by mutation operator and reduce the extra repair work, three mutation operators are designed in the paper.

#### Swap_in_Critical_Factory

Select two jobs from $\pi^{{F_{*} }}$ randomly and exchange the positions of two jobs.

#### Inverse_in_Critical_Factory

Select two jobs from $\pi^{{F_{*} }}$ randomly and inverse the jobs between the selected two jobs.

#### Insert_among_Factories

Remove a job from $\pi^{{F_{*} }}$ and reinsert it into the position selected from one of its available factories *F*_*r*_ randomly.

The above mutation operators can be executed for 15 cycles at most in the paper. If $$\pi^{^{\prime}}$$ is generated by the one of the mutation operators performed on the parent food source *π* in a certain cycle. If *f*(*π*^*’*^)$$\prec$$ *f*(*π*), then *π* $$\leftarrow$$$$\pi^{^{\prime}}$$, *limit* = 0 and the cycle is terminated If the *f*($$\pi^{^{\prime}}$$) and *f*(*π*) do not dominate each other, then *f*($$\pi^{^{\prime}}$$) will be compared with other individuals in the populations (denotes as $$\pi^{^{\prime\prime}}$$) to see if there is a dominant relationship between them. If there is a dominant relationship, the dominant solution will be abandoned and continue with next cycle. If there is no solution that can dominate *f*(*π*) at the end of 15 cycles, then *limit* = *limit* + 1. The pseudocode of the mutation operators is as follows, and F_*_ is the critical factory with the greatest makespan among all factories.
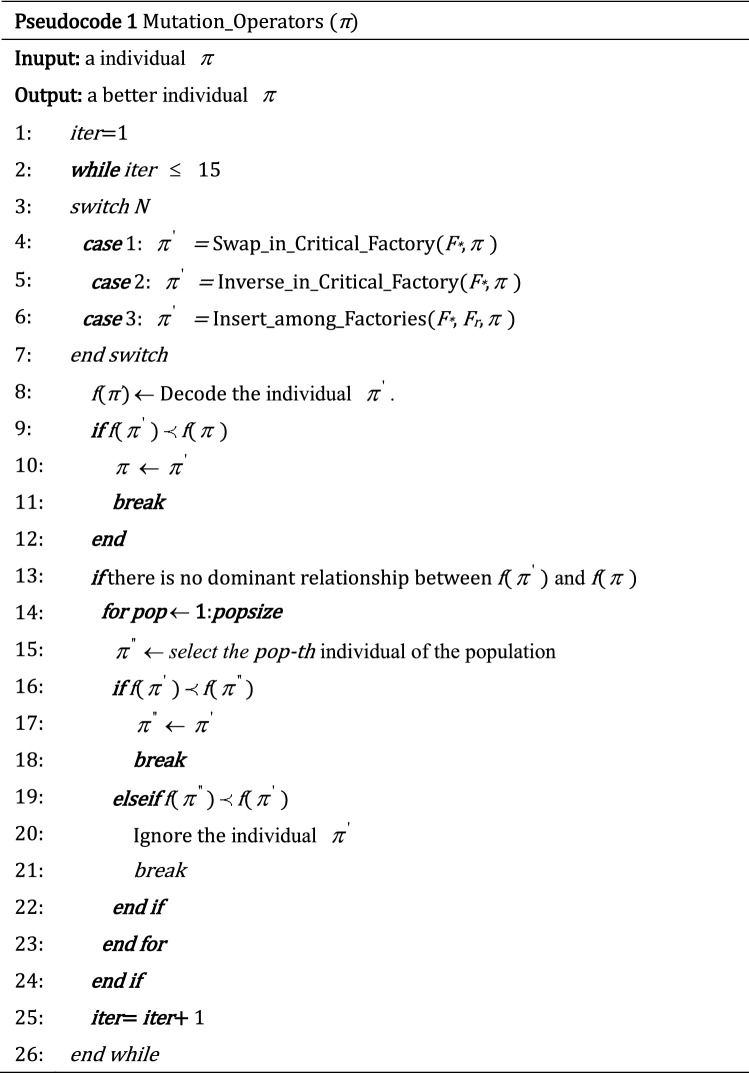


### Onlooker bee phase

To reduce the makespan, it is necessary to readjust the factory assignment and job sequence between the non-critical factory and critical factory or within the critical factory. Based on the theory of the critical factory and the features of the problem, the neighborhood search method is used in the onlooker bee phase, which consists of three kinds of neighborhood structures.

#### Greedy_Insert_in_Critical_Factory

Remove a job from $\pi^{{F_{*} }}$ randomly and reinsert it into all possible position of the original jobs sequence of factory F_*_. The original solution is replaced if the neighboring solution is better than it.

#### Greedy_Swap_in_Critical_Factory

Select a job from $\pi^{{F_{*} }}$ randomly and swap it with the rest jobs of factory F_*_ one by one. The original solution is replaced if the neighboring solution is better than it.

#### Greedy_Insert_among_Factories

Remove a job from $\pi^{{F_{*} }}$ randomly and reinsert it into all possible position in the rest of its available factories. The original solution is replaced if the neighboring solution is better than it.
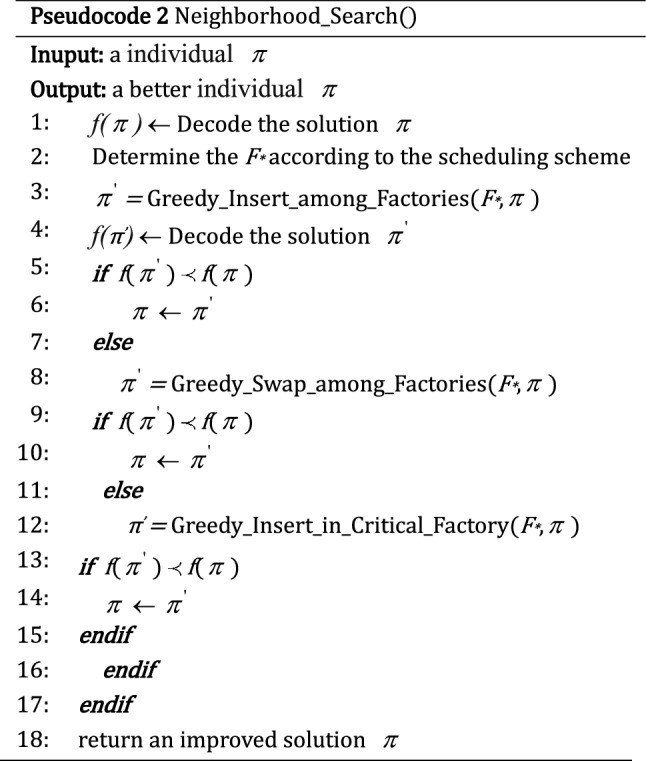


### Scout bee phase

For the MOABC, if the fitness value of the employed bee in the same food source is not improved by more than *limit* times searches, the employed bee is converted to a scout bee. In such a situation, a new individual will replace the old food source. To be specific, the process of obtaining new food sources is as follows: firstly, select a food source from the external archives randomly; then, the mutation operator proposed in this paper is used to explore near the food source; lastly, get the new food source after the mutation operator.

## Simulation experiment

Since there are few studies on the DHRHFSP-SDST, three representative algorithms NSGA-II^26^, MO-GVNS^27^ and SPEA-II are selected for comparative study. These three algorithms are widely used in solving mixed flow shop scheduling problems. In addition, MATLAB R2017a is used in this paper implements algorithm programming.

### Test instances and parameter settings

The test instances in this paper are named using f*j*s*r*, where * is a positive integer. For example, the instance f2j15s3r1 represents that 15 jobs are processed in 2 factories with 3 stages and 1 re-entrance. According to the combination of the number of jobs, stages and re-entrance with different scales, 48 examples are generated. The value ranges of all variables are shown in Table 5. The TOU price function is shown in Table 4. It's easy to observe that the on-peak price is over four times higher than the off-peak price. With such a vast disparity, energy-intensive industrial companies may save a significant amount of energy consumption costs.

**Table 5:** Bounds of the variables.

Variables	Bounds
*F*	2, 3, 4
*n*	15, 20, 25, 30
*s*	3, 4
*r*	1, 2
$m_{if}$	1, 2, 3
*PW*_*q*_	A real number between [9–15]
*PS*_*q*_	A real number between [2–4]
*PI*_*q*_	A real number between [1–1.8]
$p_{jkuif}$	An integer between [1–10]
$u_{jhuif}$	An integer between [1–6]

The key parameters in MOABC are population size *Ps*, crossover probability *Pc* and mutation probability *Pm*. Taguchi method^28^ is used to do the orthogonal experiment. The factor level of each parameter is shown in Table 6. According to the L16(4^3^) orthogonal table, each combination parameters in the orthogonal table is run 20 times independently. In the paper, the *limit* is 5 and max iterations is 120 for each algorithm. The average value of the dominant indicator Ω of 10 randomly selected cases represents the response variable (RV), as shown in Table 7, where Ω refers to the probability that the non-dominated solution set of an algorithm is optimal pareto fronts (OPF) at the same time. The greater the RV value, the better the performance of the parameter combination.

**Table 6:** Parameters levels.

Parameters	Factor level			
	1	2	3	4
*Ps*	60	80	100	120
*Pc*	0.6	0.7	0.80	0.9
*Pm*	0.1	0.15	0.2	0.25

**Table 7:** Orthogonal matrix and RV values.

Number	Factor			RV
	*Ps*	*Pc*	*Pm*	
1	60	0.7	0.15	0.0644
2	60	0.8	0.20	0.0417
3	60	0.9	0.25	0.0489
4	80	0.6	0.15	0.0676
5	80	0.7	0.10	0.0837
6	80	0.8	0.25	0.0575
7	80	0.9	0.20	0.0563
8	100	0.6	0.20	0.0800
9	100	0.7	0.25	0.0981
10	100	0.8	0.10	0.0766
11	100	0.9	0.15	0.0704
12	120	0.6	0.25	0.0476
13	120	0.7	0.20	0.0676
14	120	0.8	0.15	0.0728
15	120	0.9	0.10	0.0333
16	60	0.7	0.15	0.0644

**Figure 13:**
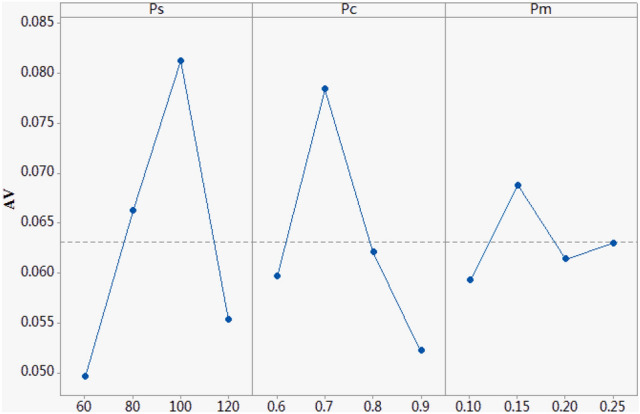
Factor level trend.

The larger value of *Ps* can promote the exploration capability but results in huge computation time. An appropriate *Pc* can improve the global search ability of the algorithm, and an appropriate *Pm* can accelerate the convergence of the algorithm to the optimal solution, and will not cause the solutions close to the optimal solution to be destroyed due to mutation. According to Fig. 13, the parameter values are set as follows: *Ps* = 100, *Pc* = 0.7 and *Pm* = 0.15, the algorithm has the best performance.

### Performance metrics

Two performance metrics IGD and C-metric in literature^29^ are used to measure the convergence and coverage of the algorithms. The smaller the IGD values, the better the algorithm's performance. On the contrary, the larger the C-metric value, the better the algorithm's performance. Furthermore, because the test instances' OPF are unknown, we substitute approximate Pareto-front (APF) for OPF. For each instance, APF may aggregate the non-dominant solutions acquired by all algorithms into a set and then eliminate the dominant solutions from the set.

### Effectiveness of energy cost saving operator based on the right shift

MOABC is compared with MOABC1 (MOABC without energy-saving operator based on the right shift) to verify the effectiveness of the energy saving operator. 12 instances are selected randomly, and the average values of IGD as well as C-metric obtained from 20 runs of each instance are listed in Table 8. The optimal value for each metric is shown in bold. Meanwhile, the Wilcoxon signed rank sum test results are given in Table 8, and the significance level is 0.05, in which "†" represents that MOABC algorithm is significantly better than the comparison algorithm, "‡" represents that MOABC algorithm is significantly worse than the comparison algorithm, and " = " represents that MOABC algorithm has no significant difference with the comparison algorithm. As can be seen from Table 8, in terms of C-metric, the two algorithms have significant differences, and MOABC is better. The IGD metric of MOABC is smaller than that of MOABC1 in all instances. Therefore, the energy saving operator designed in this paper makes full use of the characteristics of the problem and the TOU price policy to improve the individual, which is very effective.

**Table 8:** Comparison of three indexes of MOABC and MOABC1 algorithm.

Instances	IGD		C-metric	
	MOABC	MOABC1	C(MOABC, MOABC1)	C(MOABC1, MOABC)
f2j15s3r1	**0.0537†**	0.2828	**1†**	0
f2j15s4r2	**0.1393†**	0.1802	**0.8823†**	0.0321
f2j20s3r1	**0.0227†**	0.2484	**1†**	0
f2j20s4r2	**0.0484†**	0.2605	**1†**	0
f2j25s3r1	**0.0730†**	0.1674	**1†**	0
f2j25s4r2	**0.0487†**	0.2717	**0.8576†**	0.0516
f2j30s3r1	**0.0598†**	0.1836	**1†**	0
f2j30s4r2	**0.0781†**	0.3569	**1†**	0
f3j25s3r1	**0.1048†**	0.1908	**1†**	0
f3j25s4r2	**0.0614†**	0.2888	**1†**	0
f3j30s3r1	**0.0771†**	0.1791	**1†**	0
f3j30s4r2	**0.0915†**	0.3021	**1†**	0

**Figure 14:**
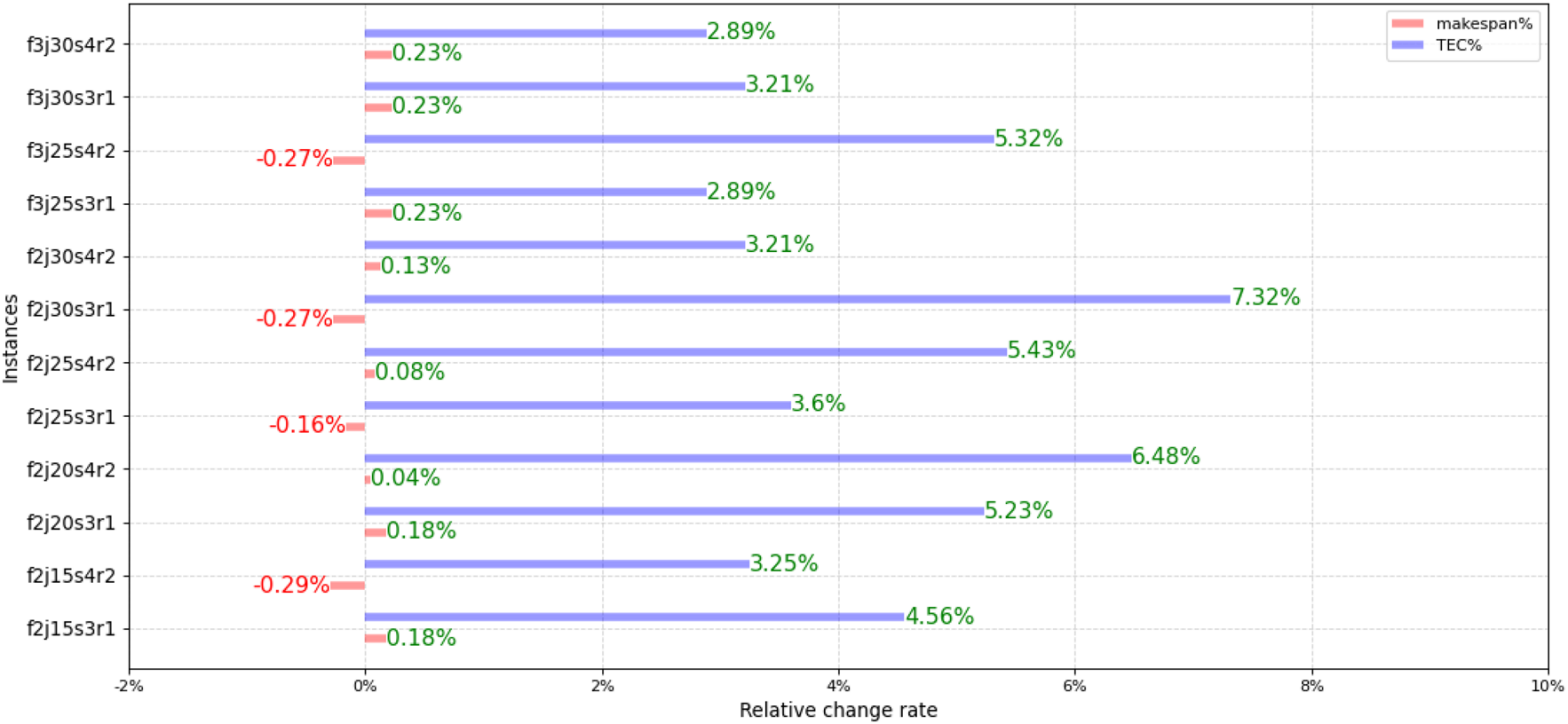
The relative change rate.

The relative change rate of the average value of *C*_*max*_ and *TC* about each instance is shown in Fig. 14, where the expressions of the relative change rate are shown in (20) and (21). $makespan_{{}}^{{{\text{MOABC1}}}}$ and $TC^{{{\text{MOABC1}}}}$ represent the average value of the two objective function values obtained by MOABC1, $makespan_{{}}^{{{\text{MOABC}}}}$ and $TC^{{{\text{MOABC}}}}$ represent the average value of the two objective function values obtained by MOABC. Meanwhile, the negative sign represents the relative increase. As can be seen from Fig. 14, after adding the energy saving operator, *TC* changes greatly, while the influence on makespan is small and can be ignored. which further proves the effectiveness of the energy saving operator.

20 $$makespan{\text{\% }} = (makespan^{{{\text{MOABC1}}}} { - }makespan^{{{\text{MOABC}}}} )/makespan^{{{\text{MOABC1}}}} {*}100{\text{\% }}$$

21 $$TC{\text{\% = }}(TC^{{{\text{MOABC1}}}} { - }TC^{{{\text{MOABC}}}} )/TC^{{{\text{MOABC1}}}} {*}100{\text{\% }}$$

### Effectiveness of population initialization

MOABC is compared with MOABC2, which generate the initial population completely randomly, to verify the effectiveness of hybrid population initialization strategy. 12 instances are selected randomly, and the average values of IGD as well as C-metric obtained from 20 runs of each instance are listed in Table 9. The best value for each metric is highlighted in bold. Table 9 shows that two algorithms have significant differences in terms of C-metric, and MOABC is better. The IGD metric of MOABC is smaller than that of MOABC2 in all instances except the f2j15s4r2. It demonstrates that the initialization strategy helps to improve the algorithm's performance.

**Table 9:** Comparison of three indexes of MOABC and MOABC2 algorithm.

Instances	IGD		C-metric	
	MOABC	MOABC2	C(MOABC, MOABC2)	C(MOABC2, MOABC)
f2j15s3r1	**0.0618†**	0.4163	**0.6684†**	0.1032
f2j15s4r2	**0.1344†**	0.3383	**0.7942†**	0.1382
f2j20s3r1	**0.0899†**	0.3973	**0.6752†**	0.2635
f2j20s4r2	**0.1062†**	0.3251	**0.5896†**	0.3465
f2j25s3r1	**0.0590†**	0.2918	**0.6365†**	0.2943
f2j25s4r2	**0.0475†**	0.2535	**0.7340†**	0.1777
f2j30s3r1	**0.0639†**	0.2880	**0.6093†**	0.1543
f2j30s4r2	**0.1023†**	0.5055	**0.6513†**	0.1636
f3j25s3r1	**0.0284†**	0.2838	**0.7219†**	0.2688
f3j25s4r2	**0.0484†**	0.1982	**0.8413†**	0.1179
f3j30s3r1	**0.0694†**	0.5174	**0.8068†**	0.1176
f3j30s4r2	**0.1505†**	0.4071	**0.5968†**	0.2244

### Comparison with other algorithms

**Figure15:**
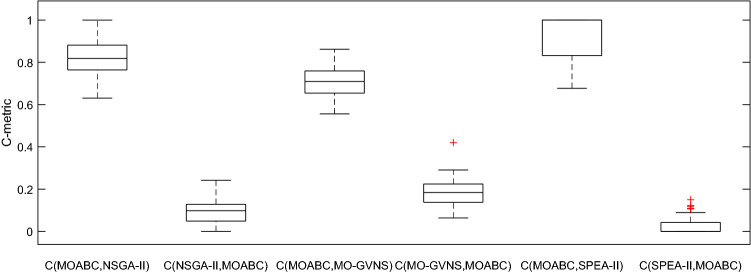
Box plot of C-metric for MOABC vs. other three algorithms.

**Figure 16:**
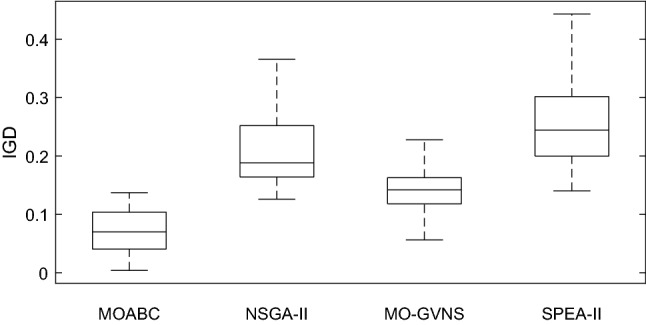
The box plot of IGD.

For the 48 instances with different scale, each algorithm is executed 20 times. Table 10 lists the average values of IGD, C-metric and the Wilcoxon signed rank test results after 20 runs of the four algorithms. It can be seen from Table 10 that except for a few instances, the IGD and C-metric indexes of MOABC are significantly better than those of NSGA-II, MO-GVNS and SPEA-II. The box plot of IGD and C-metric metrics is shown in Figs. 15 and 16. From Fig. 15, it is clear that MOABC not only has good convergence but also has uniform scalability. From Fig. 16, it can be seen that the C-metric of MOABC is much larger than other algorithms on all test instances.

**Table 10:** IGD and C-metric metrics of four algorithms (A: MOABC, B: NSGA-II, C: MO-GVNS, D: SPEA-II).

Instances	IGD				C-metric					
	MOABC	NSGA-II	MO-GVNS	SPEA-II	C(A,B)	C(B,A)	C(A,C)	C(C,A)	C(A,D)	C(D,A)
f2j15s3r1	0.0852†	0.2994	0.1746	0.3327	0.8024†	0.0435	0.6146†	0.2904	1†	0
f2j15s3r2	0.0980†	0.1802	0.1594	0.2216	0.8752	0.1143	0.7072†	0.2462	1†	0
f2j15s4r1	0.1045†	0.3170	0.1679	0.3346	0.7336†	0.0847	0.6642†	0.2024	1†	0
f2j15s4r2	0.0386†	0.2655	0.1571	0.3039	0.7847†	0.1343	0.6604†	0.1724	0.8274†	0.0504
f2j20s3r1	0.1063†	0.3657	0.2278	0.4429	1†	0	0.6012†	0.2277	1†	0
f2j20s3r2	0.0061†	0.1279	0.0965	0.1859	0.8074†	0.0932	0.7741†	0.1941	1†	0
f2j20s4r1	0.1238†	0.1882	0.1584	0.3757	0.7311†	0.1468	0.7026†	0.1849	0.8500†	0.1500
f2j20s4r2	0.0540†	0.2054	0.1231	0.2667	0.7426†	0.1575	0.6842†	0.1834	0.7784†	0.064
f2j25s3r1	0.0734†	0.1656	0.1162	0.2210	0.6930†	0.1214	0.6476†	0.1653	0.7381†	0.0225
f2j25s3r2	0.0662†	0.2360	0.1557	0.3001	0.8879†	0.1029	0.7231†	0.1421	1†	0
f2j25s4r1	0.1153 =	0.1713	0.1554	0.1843	0.8275†	0.0578	0.6472†	0.1839	0.8864†	0.0042
f2j25s4r2	0.0427†	0.1851	0.1570	0.2554	1†	0	0.8134†	0.1334	1†	0
f2j30s3r1	0.0148†	0.1883	0.1480	0.3680	0.7898†	0.1066	0.7069†	0.2254	0.8915†	0.0619
f2j30s3r2	0.0500†	0.2789	0.1286	0.3509	0.8090†	0.1098	0.7591†	0.0634	0.8468†	0.0311
f2j30s4r1	0.0330†	0.2318	0.1090	0.3031	1†	0	0.8083†	0.1039	1†	0
f2j30s4r2	0.0823†	0.2531	0.1393	0.2925	0.8256†	0.1662	0.7910†	0.0984	0.8623†	0.0388
f3j15s3r1	0.0524†	0.2638	0.1652	0.2769	0.8347†	0.0877	0.7590†	0.1109	1†	0
f3j15s3r2	0.0095†	0.2052	0.1449	0.1893	1†	0	0.8225†	0.1786	1†	0
f3j15s4r1	0.1319†	0.2377	0.1971	0.2547	0.8880†	0.0789	0.7586†	0.2228	1†	0
f3j15s4r2	0.0042†	0.1370	0.0907	0.2376	0.8646†	0.0239	0.7876†	0.1578	1†	0
f3j20s3r1	0.0457†	0.2802	0.1515	0.3062	0.7629†	0.1085	0.6253†	0.2761	1†	0
f3j20s3r2	0.0304†	0.1635	0.1268	0.2462	0.8432†	0.1288	0.8616†	0.1293	1†	0
f3j20s4r1	0.1372†	0.2803	0.2158	0.3091	0.6862†	0.0892	0.6428†	0.1955	0.8978†	0.1064
f3j20s4r2	0.0366†	0.2051	0.1890	0.2215	0.8657†	0.1173	0.7270†	0.1971	1†	0
f3j25s3r1	0.1052†	0.2157	0.1809	0.2474	0.7809†	0.1109	0.6944†	0.1817	0.7756†	0.0453
f3j25s3r2	0.0601†	0.2604	0.1603	0.2826	0.8658†	0.0644	0.7241†	0.2082	0.9019†	0.0162
f3j25s4r1	0.0450†	0.1680	0.1364	0.2107	1†	0	0.7134†	0.1760	1†	0
f3j25s4r2	0.1252 =	0.1260	0.1189	0.1494	0.7034†	0.2420	0.5557†	0.4202	0.7981†	0.0808
f3j30s3r1	0.1210†	0.3128	0.2256	0.3046	0.7802†	0.1624	0.7456†	0.2137	0.8146†	0.0226
f3j30s3r2	0.0919†	0.1724	0.1589	0.2440	1†	0	0.7584†	0.2465	1†	0
f3j30s4r1	0.0835†	0.1643	0.1221	0.2235	0.6307†	0.1701	0.6331†	0.2297	0.7226†	0.0237
f3j30s4r2	0.0809†	0.1767	0.1456	0.2023	0.7254†	0.1472	0.7008†	0.1990	0.7480†	0.1077
f4j15s3r1	0.0813†	0.2845	0.2178	0.2448	1†	0	0.7113†	0.1477	1†	0
f4j15s3r2	0.0727†	0.1818	0.1103	0.2250	0.8879†	0.1094	0.7644†	0.2016	1†	0
f4j15s4r1	0.0609†	0.1528	0.1096	0.1931	0.7305†	0.0812	0.6309†	0.0997	0.9327†	0.0278
f4j15s4r2	0.0248†	0.1540	0.0900	0.1674	0.7831†	0.1781	0.7533†	0.2355	0.8114†	0.1188
f4j20s3r1	0.1092†	0.1621	0.1174	0.2110	0.7849†	0.0534	0.7624†	0.0897	0.8192†	0.0094
f4j20s3r2	0.0436†	0.1963	0.1684	0.2479	0.8117†	0.0886	0.6466†	0.1115	1†	0
f4j20s4r1	0.0215†	0.1643	0.1295	0.1875	1†	0	0.7532†	0.1841	0.8364†	0.1220
f4j20s4r2	0.1114 =	0.1688	0.1061	0.1734	0.7653†	0.1491	0.7368†	0.2255	0.6766†	0.1098
f4j25s3r1	0.0868†	0.1508	0.1298	0.1768	0.8455†	0.0847	0.6743†	0.1328	1†	0
f4j25s3r2	0.0596†	0.1892	0.1392	0.2402	0.8664†	0.1228	0.7723†	0.2022	1†	0
f4j25s4r1	0.0045†	0.1446	0.0563	0.1402	0.7888†	0.1479	0.6619†	0.1734	1†	0
f4j25s4r2	0.0156†	0.1440	0.1067	0.2231	0.8870†	0.1065	0.6321†	0.1276	1†	0
f4j30s3r1	0.1048†	0.2510	0.1840	0.3092	0.8323†	0.0771	0.7658†	0.1056	1†	0
f4j30s3r2	0.0812†	0.1630	0.1018	0.1974	0.7123†	0.0434	0.7004†	0.2618	0.8174†	0.0892
f4j30s4r1	0.0675†	0.1520	0.1195	0.1810	0.7445†	0.0350	0.6224†	0.1795	0.8396†	0.0290
f4j30s4r2	0.1033†	0.2447	0.1355	0.2703	0.8440†	0.1268	0.6633†	0.2279	1†	0

**Figure 17:**
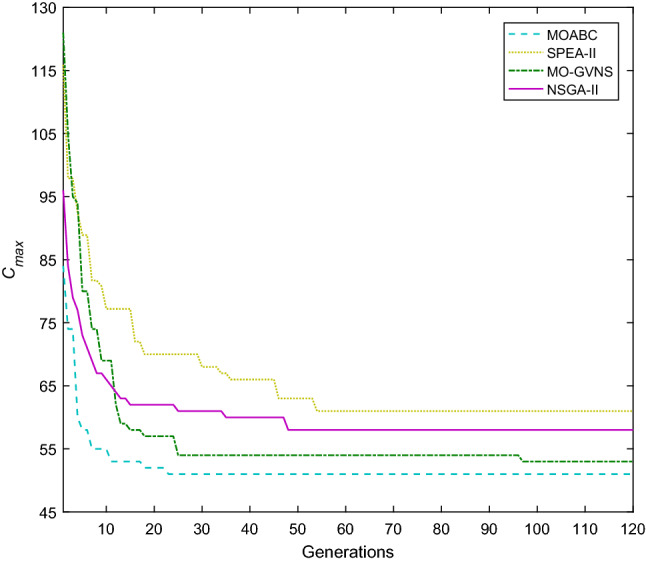
Convergence diagram of instance f2j15s3r1 about *TC.*

**Figure 18:**
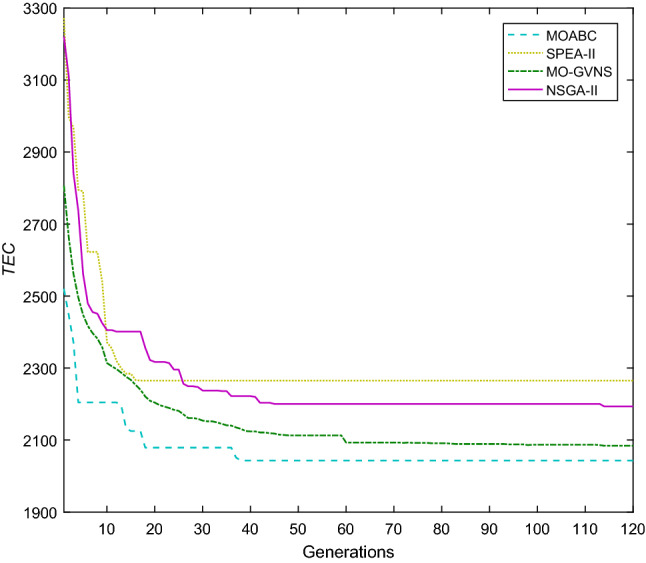
Convergence diagram of instance f2j15s3r1 about *C*_*max*_.

Taking f2j15s3r1instance as an example, in which 15 jobs are processed in 2 factories. In factory1, the number of UPMs at each stage are 2, 2 and 2. In factory2, the number of UPMs at each stage are 2, 2 and 1. The machine eligibility constraints is $F_{1} = \left\{ 2 \right\}$, $F_{2} = \left\{ 2 \right\}$, $F_{3} = \left\{ 1 \right\}$, $F_{4} = \left\{ 1 \right\}$, $F_{5} = \left\{ 1 \right\}$, $F_{6} = \left\{ {1,2} \right\}$, $F_{7} = \left\{ 1 \right\}$, $F_{8} = \left\{ 2 \right\}$, $F_{9} = \left\{ {1,2} \right\}$, $F_{10} = \left\{ {1,2} \right\}$, $F_{11} = \left\{ 1 \right\}$, $F_{12} = \left\{ 2 \right\}$, $F_{13} = \left\{ 2 \right\}$, $F_{14} = \left\{ 1 \right\}$ and $F_{15} = \left\{ {1,2} \right\}$. The convergence curves of *C*_*max*_ and *TC* for MOABC, NSGA-II, Mo-GVNS and SPEA-II algorithms are shown in Figs. 17 and 18. From the figures, we can see that two objectives of MOABC decline rapidly in the early stage of evolution, and gradually converge in the later stage, and the two goals converge to 53 h and 2050 yuan respectively, which are significantly smaller than the convergence values of other algorithms.

**Figure 19:**
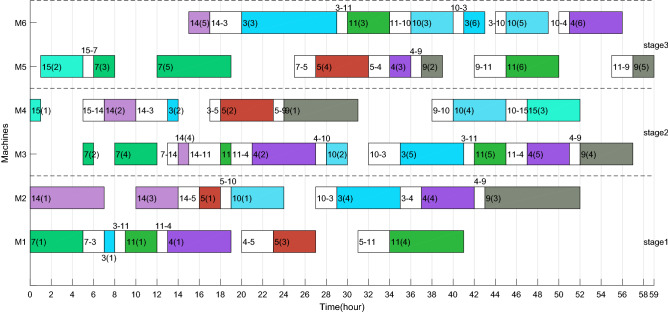
The Gantt chart of instance f2j15s3r1 in factory1 without considering TOU price.

**Figure 20:**
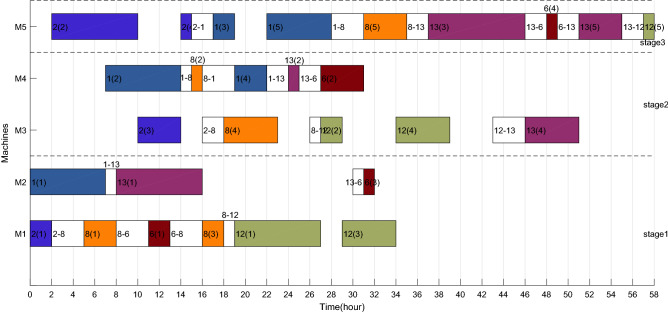
The Gantt chart of instance f2j15s3r1 in factory2 without considering TOU price.

**Figure 21:**
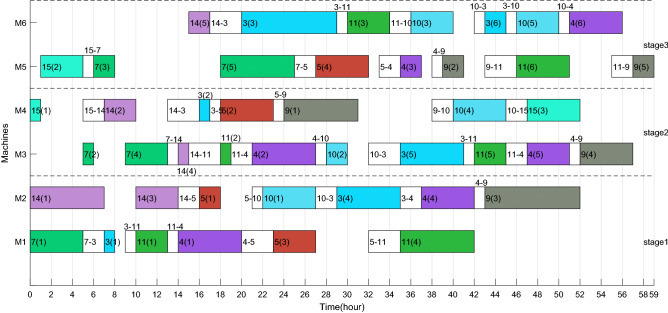
The Gantt chart of instance f2j15s3r1 in factory1 considering TOU price.

**Figure 22:**
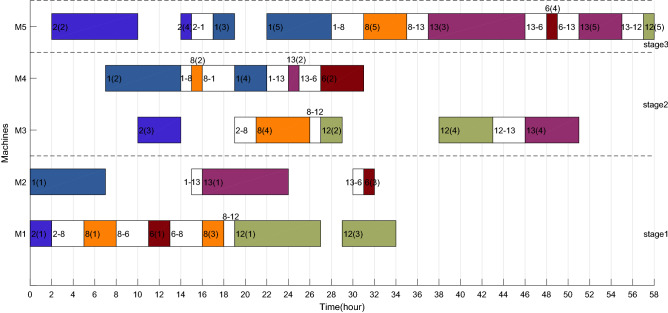
The Gantt chart of instance f2j15s3r1 in factory2 considering TOU price.

Taking the feasible solution $\pi {{ = \{ [7,14,3,5,11,4,10,9,15]}},{{[2,1,8,13,12,6]\} }}$ as an example, without considering the characteristics of TOU price, the Gantt charts of the two factories are shown in Figs. 19 and 20 respectively, and $C_{max} = max\left( {58,59} \right) = 59$ hours and *TC* = 1432.2 + 897.7 = 2329.9 yuan. Figures 21 and 22 are the Gantt charts of the solution with energy saving operator, and the two objective values are $C_{max} = max\left( {58,59} \right) = 59$ hours and *TC* = 1349.7 + 845 = 2194.7 yuan. By comparison, under the premise of keeping the production efficiency unchanged, the processing periods of many operations, such as 7(5), 4(1), 11(4), 10(1), 11(6), 9(2), 3(2), 13(1), 12(4) and 8(4), have been shifted, and TC has been reduced by 135.2, up to 5.80%. To sum up, the MOABC algorithm proposed in this paper can effectively solve DHRHFSP-SDST under TOU price.

## Conclusions and further works

The distributed re-entrant hybrid flow shop problem has attracted the attention of many scholars since it was proposed, but there are still many problems to be further studied and expanded. In the paper, we consider the distributed heterogeneous re-entrant hybrid flow shop scheduling problem with sequence dependent setup times (SDST) considering factory eligibility constraints (DHRHFSP-SDST) under time of use (TOU) price policy. According to the characteristics of this problem, a multi-objective artificial bee colony algorithm (MOABC) is proposed. The main improvements include encoding and decoding methods, energy saving operators, three neighborhood search operators and new food sources generation strategy. Finally, a large number of simulation experiments verify the effectiveness and superiority of the algorithm. Under the TOU price policy, although the total electricity consumption does not decrease, it can reasonably shift the electricity load and reduce the total cost of electricity consumption. In the future, we will further study green scheduling problems, such as designing better swarm intelligence algorithm, collaborative optimization of distributed scheduling and pre-maintenance, and so on.

## Data Availability

The datasets used and/or analysed during the current study available from the corresponding author on reasonable.
